# Crystal structure, Hirshfeld surface analysis and inter­action energy calculation of 4-(furan-2-yl)-2-(6-methyl-2,4-dioxo­pyran-3-yl­idene)-2,3,4,5-tetra­hydro-1*H*-1,5-benzodiazepine

**DOI:** 10.1107/S2056989021007441

**Published:** 2021-07-27

**Authors:** Mohamed El Hafi, Sanae Lahmidi, Lhoussaine El Ghayati, Tuncer Hökelek, Joel T. Mague, Bushra Amer, Nada Kheira Sebbar, El Mokhtar Essassi

**Affiliations:** a Laboratory of Heterocyclic Organic Chemistry, Department of Chemistry, Faculty of Sciences, Mohammed V University in Rabat, BP 1014, Rabat, Morocco; b Department of Physics, Hacettepe University, 06800 Beytepe, Ankara, Turkey; c Department of Chemistry, Tulane University, New Orleans, LA 70118, USA; d Faculty of Medicine and Health Sciences, Sana’a University, Sana’a, Yemen; e Applied Chemistry and Environment Laboratory, Applied Bioorganic Chemistry Team, Faculty of Science, Ibn Zohr University, Agadir, Morocco

**Keywords:** crystal structure, pyran­dione, furan, tetra­hydro­benzodiazepine, hydrogen bond, π-stacking

## Abstract

The title compound {systematic name: (*S*,*E*)-3-[4-(furan-2-yl)-2,3,4,5-tetra­hydro-1*H*-benzo[*b*][1,4]diazepin-2-yl­idene]-6-methyl-2*H*-pyran-2,4(3*H*)-dione}, C_19_H_16_N_2_O_4_, is constructed from a benzodiazepine ring system linked to furan and pendant di­hydro­pyran rings, where the benzene and furan rings are oriented at a dihedral angle of 48.7 (2)°. The pyran ring is modestly non-planar [largest deviation of 0.029 (4) Å from the least-squares plane] while the tetra­hydro­diazepine ring adopts a boat conformation. The rotational orientation of the pendant di­hydro­pyran ring is partially determined by an intra­molecular N—H_Diazp_⋯O_Dhydp_ (Diazp = diazepine and Dhydp = di­hydro­pyran) hydrogen bond. In the crystal, layers of mol­ecules parallel to the *bc* plane are formed by N—H_Diazp_⋯O_Dhydp_ hydrogen bonds and slipped π–π stacking inter­actions. The layers are connected by additional slipped π–π stacking inter­actions. A Hirshfeld surface analysis of the crystal structure indicates that the most important contributions for the crystal packing are from H⋯H (46.8%), H⋯O/O⋯H (23.5%) and H⋯C/C⋯H (15.8%) inter­actions, indicating that van der Waals inter­actions are the dominant forces in the crystal packing. Computational chemistry indicates that in the crystal the N—H⋯O hydrogen-bond energy is 57.5 kJ mol^−1^.

## Abstract 2

The pyran ring is modestly non-planar while the tetra­hydro­diazepine ring adopts a boat conformation. In the crystal, N—H⋯O hydrogen bonds and slipped π–π stacking inter­actions build a three-dimensional network structure.

## Chemical context   

1,5-Benzodiazepine derivatives are an important class of nitro­gen-containing heterocyclic compounds because of their potent biological activities, acting as anti­depressant (Sharma *et al.*, 2017 ▸), anti­tubercular (Singh *et al.*, 2017 ▸), anti­microbial (An *et al.*, 2016 ▸) and anti­convulsant agents (Jyoti & Mithlesh, 2013 ▸). Many synthetic methodologies have been developed to access this type of compound (Sebhaoui *et al.*, 2017 ▸; Chkirate *et al.*, 2018 ▸).

The present study continues the investigation of 1,5-benzodiazepine derivatives recently published by our team (El Ghayati *et al.*, 2019 ▸, 2021 ▸; Essaghouani *et al.*, 2016 ▸, 2017 ▸). In this context, we report herein the synthesis, the mol­ecular and crystal structures along with the Hirshfeld surface analysis and the inter­molecular inter­action energies of the title compound, (I). 
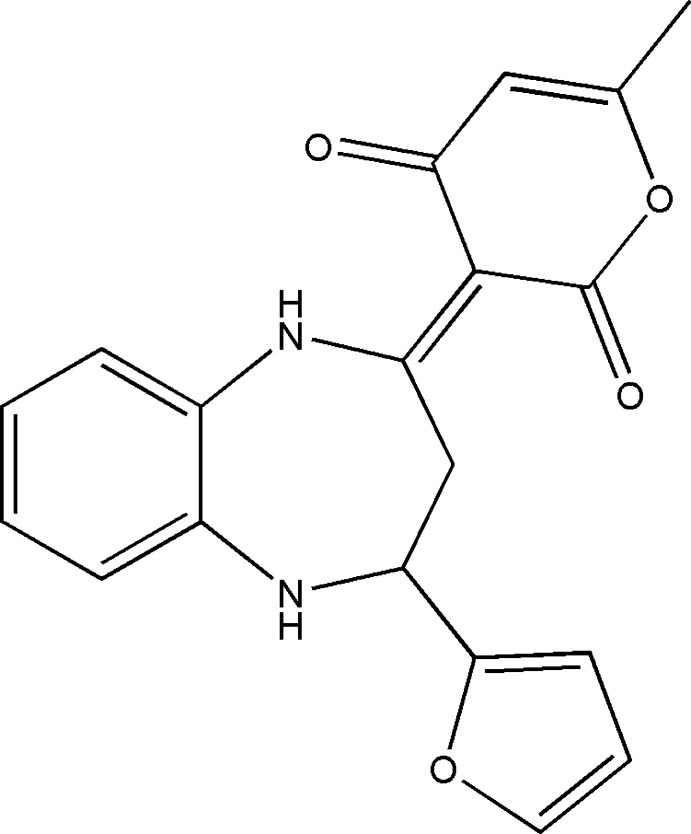


## Structural commentary   

**Figure 1:**
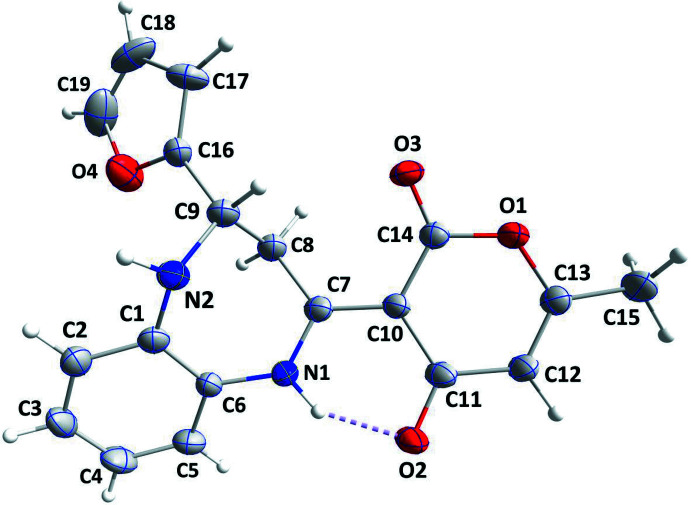
The mol­ecule of (I) with the atom-numbering scheme and displacement ellipsoids drawn at the 50% probability level. The intra­molecular hydrogen bond is depicted by a dashed line.

The O1/C10–C14 pyran ring is not planar and a puckering analysis (Cremer & Pople, 1975 ▸) yielded the parameters *Q* = 0.082 (4) Å, *θ* = 114 (3)° and *φ* = 70 (3)°, thus indicating it adopts a slightly twisted envelope conformation with C10 at the tip of the flap. In the seven-membered ring, N1 and N2 are displaced from the C1–C6 plane by 0.159 (6) and 0.158 (6) Å, respectively, in the direction away from C8 (Fig. 1 ▸). A puckering analysis of the seven-membered ring gave the parameters *Q*(2) = 0.915 (4) Å, *Q*(3) = 0.187 (4) Å, *φ*(2) = 38.9 (2)° and *φ*(3) = 156.3 (12)° [total puckering amplitude *Q* = 0.933 (4) Å]. This ring adopts a boat conformation. The mean plane of the O1/C10–C14 ring is inclined to that of the C1–C6 ring by 34.8 (1)°, while the C1–C6 and O4/C16–C19 rings make a dihedral angle of 48.7 (2)°. The orientation of the O1/C10–C14 ring is partially determined by an intra­molecular N1—H1⋯O2 hydrogen bond (Table 1 ▸, Fig. 1 ▸). All bond lengths and angles in the mol­ecule of (I) are in the expected ranges.

## Supra­molecular features   

**Figure 2:**
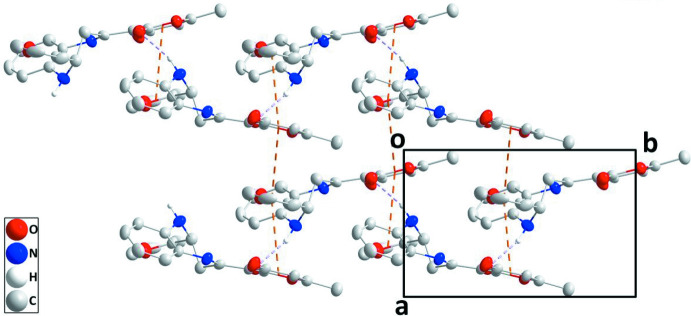
Portions of two chains viewed along the *c* axis direction with N—H⋯O hydrogen bonds and slipped π–π stacking inter­actions depicted, respectively, by violet and orange dashed lines.

**Figure 3:**
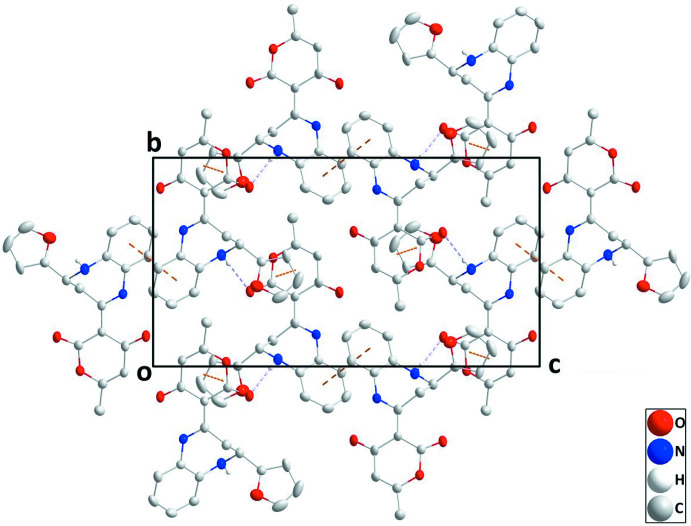
Packing viewed along the *a-*axis direction with inter­molecular inter­actions depicted as in Fig. 2 ▸.

In the crystal, N—H_Diazp_⋯O_Dhydp_ (Diazp = diazepine and Dhydp = di­hydro­pyran) hydrogen bonds (Table 1 ▸) form helical chains of mol­ecules extending along the *b-*axis direction. The chains are reinforced by slipped π–π stacking inter­actions between furan and pyran rings within the chains [centroid⋯centroid(−*x* + 1, *y* + 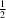 , −*z* + 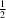 ) distance = 3.610 (2) Å, dihedral angle = 4.4 (2)°, slippage = 1.14 Å]. The chains are connected into layers parallel to the *bc* plane by analogous π–π stacking inter­actions (Fig. 2 ▸) [centroid⋯centroid(−*x* + 1, *y* − 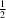 , −*z* + 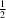 ) distance = 3.610 (2) Å, dihedral angle = 4.4 (2)°, slippage = 1.38 Å]. The layers are connected by slipped π–π stacking inter­actions between inversion-related C1–C6 rings [centroid⋯centroid (−*x* + 1, −*y*, −*z* + 1) distance = 3.690 (2) Å, slippage = 1.47 Å] (Fig. 3 ▸).

## Hirshfeld surface analysis   

**Figure 4:**
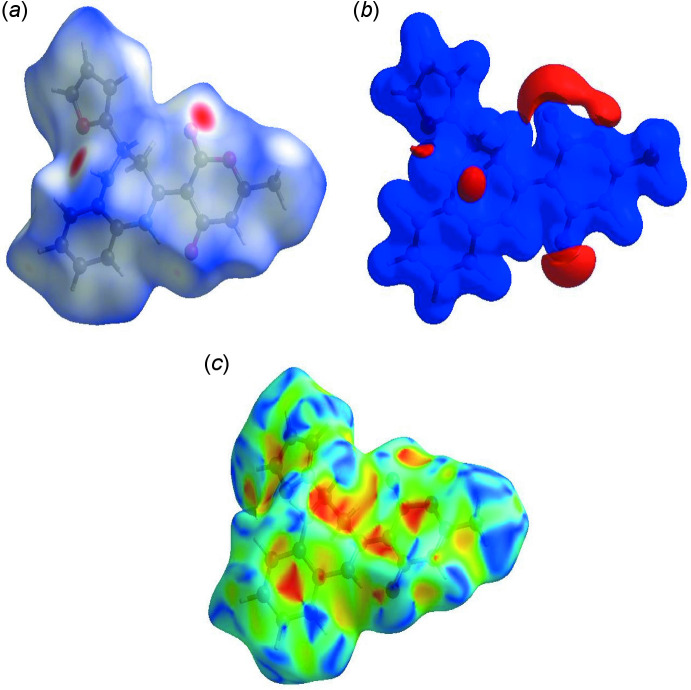
(*a*) View of the three-dimensional Hirshfeld surface of the title compound, plotted over *d*_norm_ in the range of −0.3842 to 1.4934 a.u., (*b*) view of the three-dimensional Hirshfeld surface of the title compound plotted over electrostatic potential energy in the range −0.0500 to 0.0500 a.u. using the STO-3 G basis set at the Hartree–Fock level of theory and (*c*) Hirshfeld surface of the title compound plotted over shape-index.

**Figure 5:**
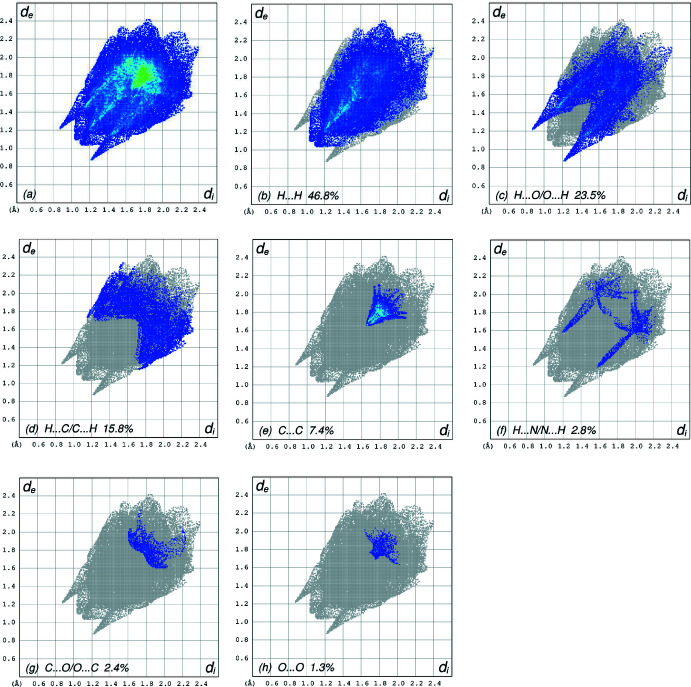
The full two-dimensional fingerprint plots for the title compound, showing (*a*) all inter­actions, and delineated into (*b*) H⋯H, (*c*) H⋯O/O⋯H, (*d*) H⋯C/C⋯H, (*e*) C⋯C, (*f*) H⋯N/N⋯H, (*g*) C⋯O/O⋯C and (*h*) O⋯O inter­actions. The *d*_i_ and *d*_e_ values are the closest inter­nal and external distances (in Å) from given points on the Hirshfeld surface.

In order to visualize the inter­molecular inter­actions in the crystal of (I), a Hirshfeld surface (HS) analysis (Hirshfeld, 1977 ▸) was carried out using *Crystal Explorer 17.5* (Turner *et al.*, 2017 ▸). In the HS plotted over *d*_norm_ (Fig. 4 ▸ *a*), the white surface indicates contacts with distances equal to the sum of van der Waals radii, and the red and blue colours indicate distances shorter or longer than the van der Waals radii, respectively (Venkatesan *et al.*, 2016 ▸). The bright-red spots appearing near O3 and hydrogen atom H2*A* indicate their roles as the respective donor and/or acceptor atoms in hydrogen bonding. They also appear as blue and red regions corres­ponding to positive and negative potentials on the HS mapped over electrostatic potential (Spackman *et al.*, 2008 ▸; Jayatilaka *et al.*, 2005 ▸) as shown in Fig. 4 ▸ *b*. The blue regions indicate the positive electrostatic potential (hydrogen-bond donors), while the red regions indicate the negative electrostatic potential (hydrogen-bond acceptors). The shape-index of the HS is a tool to visualize the π–π stacking by the presence of adjacent red and blue triangles. Fig. 4 ▸ *c* clearly suggests that there are π–π inter­actions in (I). The overall two-dimensional fingerprint plot, Fig. 5 ▸ *a*, and those delineated into H⋯H, H⋯O/O⋯H, H⋯C/C⋯H, C⋯C, H⋯N/N⋯H, C⋯ O/O⋯C and O⋯O contacts (McKinnon *et al.*, 2007 ▸) are illustrated in Fig. 5 ▸ *b*–*h*, respectively, together with their relative contributions to the Hirshfeld surface. The most important inter­action is H⋯H (Table 2 ▸) contributing 46.8% to the overall crystal packing, which is reflected in Fig. 5 ▸ *b* as widely scattered points of high density due to the large hydrogen content of the mol­ecule with the tip at *d*_e_ = *d*_i_ = 1.07 Å. The pair of scattered points of spikes in the fingerprint plot delineated into H⋯O/O⋯H contacts (23.5% contribution to the HS,Fig. 5 ▸ *c*; Table 2 ▸) have the tips at *d*_e_ + *d*_i_ = 2.09 Å. In the absence of C—H⋯π inter­actions, the pair of characteristic wings in the fingerprint plot delineated into H⋯C/C⋯H contacts (Fig. 5 ▸ *d*, 15.8%) have tips at *d*_e_ + *d*_i_ = 2.95 Å. The C⋯C contacts (Fig. 5 ▸ *e*, 7.4%) have an arrow-shaped distribution of points with its tip at *d*_e_ = *d*_i_ = 1.65 Å. The H⋯N/N⋯H contacts (Fig. 5 ▸ *f*, 2.8%) have tips at *d*_e_ + *d*_i_ = 2.78 Å. Finally, the C⋯O/O⋯C (Fig. 5 ▸ *g*) and O⋯O (Fig. 5 ▸ *h*) contacts (2.4% and 1.3% contributions, respectively, to the HS) appear with tips at *d*_e_ + *d*_i_ = 3.50 Å and *d*_e_ = *d*_i_ = 1.73 Å, respectively.

**Figure 6:**
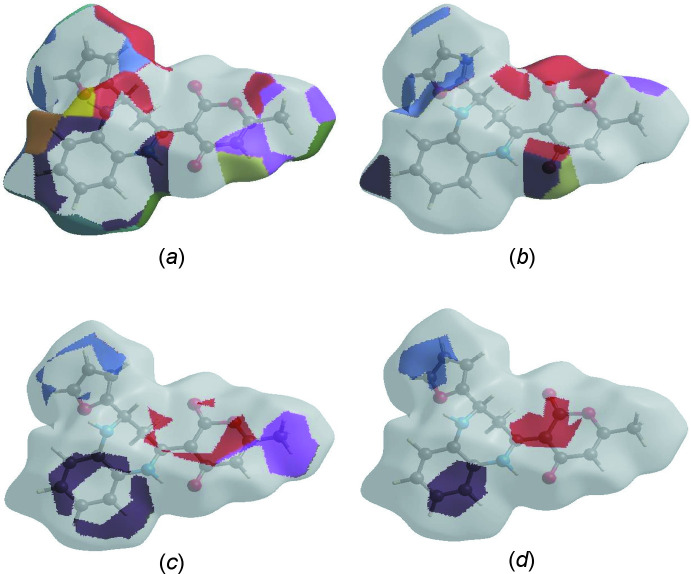
The Hirshfeld surface representations with the function *d*_norm_ plotted onto the surface for (*a*) H⋯H, (*b*) H⋯O/O⋯H, (*c*) H⋯C/C⋯H and (*d*) C⋯C inter­actions.

The Hirshfeld surface representations with the function *d*_norm_ plotted onto the surface are shown for the H⋯H, H⋯O/O⋯H, H⋯C/C⋯H and C⋯C inter­actions in Fig. 6 ▸ *a*–*d*, respectively.

The Hirshfeld surface analysis confirms the importance of H-atom contacts in establishing the packing. The large number of H⋯H, H⋯O/O⋯H, and H⋯C/C⋯H inter­actions suggest that van der Waals inter­actions play the major role in the crystal packing (Hathwar *et al.*, 2015 ▸).

## Inter­action energy calculations   

The inter­molecular inter­action energies were calculated using the CE–B3LYP/6–31G(d,p) energy model available in *Crystal Explorer 17.5* (Turner *et al.*, 2017 ▸), where a cluster of mol­ecules is generated by applying crystallographic symmetry operations with respect to a selected central mol­ecule within the default radius of 3.8 Å (Turner *et al.*, 2014 ▸). The total inter­molecular energy (*E*_tot_) is the sum of electrostatic (*E*_ele_), polarization (*E*_pol_), dispersion (*E*_dis_) and exchange-repulsion (*E*_rep_) energies (Turner *et al.*, 2015 ▸) with scale factors of 1.057, 0.740, 0.871 and 0.618, respectively (Mackenzie *et al.*, 2017 ▸). The hydrogen bonding inter­action energy for the N2—H2*A*⋯O3 hydrogen bond was calculated (in kJ mol^−1^) as −32.6 (*E*_ele_), −7.4 (*E*_pol_), −60.8 (*E*_dis_), 57.3 (*E*_rep_) and −57.5 (*E*_tot_).

## Database survey   

**Figure 7:**
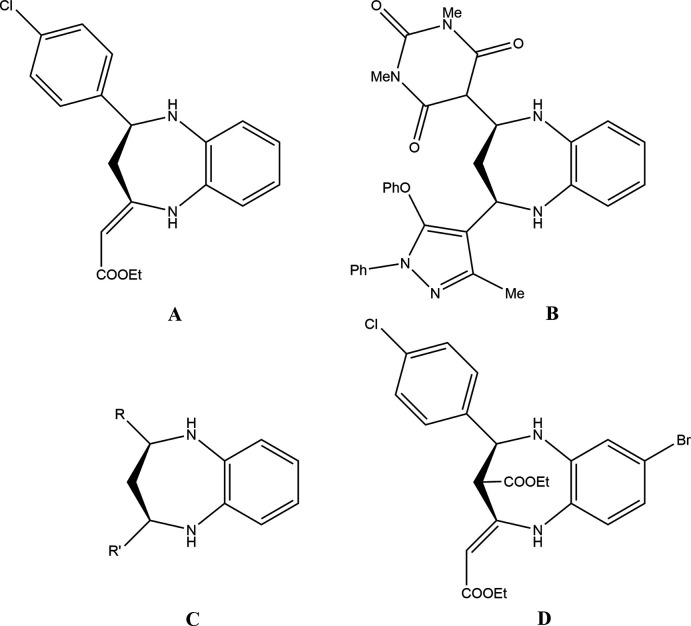
Diagrams of compounds structurally related to (I).

A search of the Cambridge Structural Database (CSD, updated 29 May 2021; Groom *et al.*, 2016 ▸) for 2,3,4,5-tetra­hydro-1*H*benzo[*b*][1,4] diazepines substituted at the 2- and 4-positions gave a substantial number of hits with seven deemed closely similar to the title mol­ecule (Fig. 7 ▸). These are: **A** (Lal *et al.*, 2013 ▸), **B** (Siddiqui & Siddiqui, 2020 ▸), **C** with *R* = 4-ClC_6_H_4_, thio­phene, 3,4-(MeO)C_6_H_3_ and *R*′ = 6- methyl-2*H*-pyran-2,4-(3*H*)-dione as well as *R* = 6-methyl-2*H*- pyran-2,4-(3*H*)-dione and *R*′ = 3-BrC_6_H_4_ (Faidallah *et al.*, 2015 ▸) and **D** (Wu & Wang, 2020 ▸) (Fig. 7 ▸). All have the tetra­hydro­diazepine ring adopting a boat conformation with puckering amplitudes in the range 0.702 (2) Å (for **A**) to 0.957 (2) Å (for **C**, *R* = thio­phene). The dihedral angles between the mean planes of the benzo rings and those of the ring-containing substituents on the seven-membered ring vary considerably, likely due to packing considerations as the steric bulk of these groups differ markedly.

## Synthesis and crystallization   

To a suspension of 3-[1-(2-amino­phenyl­imino)­eth­yl]-4-hy­droxy-6-methyl­pyran-2-one (4 mmol) in ethanol (40 ml) were added 1.5 equivalents of furan-2-carboxaldehyde and four drops of tri­fluoro­acetic acid (TFA). The mixture was refluxed for 3 h. Cooling to room temperature induced the precipitation of a yellow solid, which was filtered off, and then washed with 20 ml of cold ethanol. Crystals suitable for X-ray analysis were obtained by recrystallization of the bulk from ethanol solution to afford colourless crystals (yield: 75%).

## Refinement   

Crystal, data collection and refinement details are presented in Table 3 ▸. Inspection of the data with *CELL_NOW* (Sheldrick, 2009 ▸) revealed that the crystal under investigation was twinned by a 180° rotation about the a* axis with a subsequently refined 78:22 ratio of the two twin components. The full two-component reflection file (HKLF-5 format) was used for the final refinement. Hydrogen atoms attached to carbon were included as riding contributions in idealized positions (C—H = 0.95–0.99 Å) with *U*_iso_(H) = 1.2–1.5*U*_eq_(C). Those attached to nitro­gen were restrained to a target bond length of 0.91 Å using the DFIX instruction in *SHELXL*. The displacement ellipsoids of the O1/C10–C14 ring suggest a possible slight disorder in this group, but it does not appear large enough to model with alternate locations of the atoms.

## Supplementary Material

Crystal structure: contains datablock(s) I, global. DOI: 10.1107/S2056989021007441/wm5612sup1.cif

Supporting information file. DOI: 10.1107/S2056989021007441/wm5612Isup3.cdx

Structure factors: contains datablock(s) I. DOI: 10.1107/S2056989021007441/wm5612Isup4.hkl

Supporting information file. DOI: 10.1107/S2056989021007441/wm5612Isup4.cml

CCDC reference: 2097593

Additional supporting information:  crystallographic information; 3D view; checkCIF report

## Figures and Tables

**Table 1:** Hydrogen-bond geometry (Å, °)

*D*—H⋯*A*	*D*—H	H⋯*A*	*D*⋯*A*	*D*—H⋯*A*
N1—H1⋯O2	0.91 (1)	1.72 (3)	2.538 (4)	148 (4)
N2—H2*A*⋯O3^vi^	0.91 (1)	2.20 (2)	3.079 (4)	162 (5)

Symmetry code: (vi) -x+1, y-{\script{1\over 2}}, -z+{\script{1\over 2}}.

**Table 2:** Selected interatomic distances (Å)

O2⋯N1	2.537 (4)	N1⋯N2	2.865 (4)
O3⋯C8	2.856 (4)	C4⋯C6^iii^	3.387 (5)
O3⋯N2^i^	3.079 (4)	C14⋯C16^i^	3.407 (5)
O4⋯N2	2.955 (5)	C1⋯H8*A*	2.68
O2⋯H12^ii^	2.74	C6⋯H8*A*	2.59
O2⋯H3^iii^	2.62	C11⋯H1	2.28 (3)
O2⋯H1	1.72 (3)	C14⋯H2*A* ^i^	2.79 (4)
O3⋯H2*A* ^i^	2.20 (4)	C14⋯H8*B*	2.64
H15*C*⋯O3^iv^	2.70	H2⋯H2*A*	2.29
O3⋯H2^i^	2.68	H2⋯H17^vi^	2.33
O3⋯H8*B*	2.23	H3⋯H17^vi^	2.38
O4⋯H15*B* ^v^	2.70	H12⋯H15*A*	2.42

Symmetry codes: (i) -x+1, y+{\script{1\over 2}}, -z+{\script{1\over 2}}; (ii) -x+2, -y+1, -z+1; (iii) -x+1, -y, -z+1; (iv) -x+2, y+{\script{1\over 2}}, -z+{\script{1\over 2}}; (v) x, y-1, z; (vi) -x+1, y-{\script{1\over 2}}, -z+{\script{1\over 2}}.

**Table 3:** Experimental details

Crystal data	
Chemical formula	C_19_H_16_N_2_O_4_
*M*_r_	336.34
Crystal system, space group	Monoclinic, *P*2_1_/*c*
Temperature (K)	150
*a*, *b*, *c* (Å)	7.0111 (8), 11.0123 (13), 20.493 (2)
β (°)	96.202 (5)
*V* (Å^3^)	1573.0 (3)
*Z*	4
Radiation type	Mo *K*α
μ (mm^−1^)	0.10
Crystal size (mm)	0.34 × 0.22 × 0.11

Data collection	
Diffractometer	Bruker D8 QUEST PHOTON 3 diffractometer
Absorption correction	Multi-scan (*TWINABS*; Sheldrick, 2009 ▸)
*T*_min_, *T*_max_	0.97, 0.99
No. of measured, independent and observed [*I* > 2σ(*I*)] reflections	5201, 5201, 4007
*R*_int_	0.081
(sin θ/λ)_max_ (Å^−1^)	0.672

Refinement	
*R*[*F* ^2^ > 2σ(*F* ^2^)], *wR*(*F* ^2^), *S*	0.079, 0.214, 1.14
No. of reflections	5201
No. of parameters	236
No. of restraints	2
H-atom treatment	H atoms treated by a mixture of independent and constrained refinement
Δρ_max_, Δρ_min_ (e Å^−3^)	0.54, −0.30

Computer programs: *APEX3* and *SAINT* (Bruker, 2020 ▸), *SHELXT* (Sheldrick, 2015*a* ▸), *SHELXL2018/1* (Sheldrick, 2015*b* ▸), *DIAMOND* (Brandenburg & Putz, 2012 ▸) and *publCIF* (Westrip, 2010 ▸).

## supplementary crystallographic information

### Crystal data

**Table:**

C_19_H_16_N_2_O_4_	*F*(000) = 704
*M**_r_* = 336.34	*D*_x_ = 1.420 Mg m^−^^3^
Monoclinic, *P*2_1_/*c*	Mo *K*α radiation, λ = 0.71073 Å
*a* = 7.0111 (8) Å	Cell parameters from 9980 reflections
*b* = 11.0123 (13) Å	θ = 2.7–28.4°
*c* = 20.493 (2) Å	µ = 0.10 mm^−^^1^
β = 96.202 (5)°	*T* = 150 K
*V* = 1573.0 (3) Å^3^	Block, colourless
*Z* = 4	0.34 × 0.22 × 0.11 mm

### Data collection

**Table:**

Bruker D8 QUEST PHOTON 3 diffractometer	5201 independent reflections
Radiation source: fine-focus sealed tube	4007 reflections with *I* > 2σ(*I*)
Graphite monochromator	*R*_int_ = 0.081
Detector resolution: 7.3910 pixels mm^-1^	θ_max_ = 28.5°, θ_min_ = 2.7°
φ and ω scans	*h* = −9→9
Absorption correction: multi-scan (*TWINABS*; Sheldrick, 2009)	*k* = 0→14
*T*_min_ = 0.97, *T*_max_ = 0.99	*l* = 0→27
5201 measured reflections	

### Refinement

**Table:**

Refinement on *F*^2^	Primary atom site location: dual
Least-squares matrix: full	Secondary atom site location: difference Fourier map
*R*[*F*^2^ > 2σ(*F*^2^)] = 0.079	Hydrogen site location: mixed
*wR*(*F*^2^) = 0.214	H atoms treated by a mixture of independent and constrained refinement
*S* = 1.14	*w* = 1/[σ^2^(*F*_o_^2^) + (0.0648*P*)^2^ + 2.9838*P*] where *P* = (*F*_o_^2^ + 2*F*_c_^2^)/3
5201 reflections	(Δ/σ)_max_ < 0.001
236 parameters	Δρ_max_ = 0.54 e Å^−^^3^
2 restraints	Δρ_min_ = −0.30 e Å^−^^3^

### Special details

**Table:**

Experimental. The diffraction data were obtained from 9 sets of frames, each of width 0.5° in ω or φ, collected with scan parameters determined by the "strategy" routine in *APEX3*. The scan time was 20 sec/frame. Analysis of 2110 reflections having I/σ(I) > 15 and chosen from the full data set with *CELL_NOW* (Sheldrick, 2008) showed the crystal to belong to the monoclinic system and to be twinned by a 180° rotation about the *a* axis. The raw data were processed using the multi-component version of *SAINT* under control of the two-component orientation file generated by *CELL_NOW*.
Geometry. All esds (except the esd in the dihedral angle between two l.s. planes) are estimated using the full covariance matrix. The cell esds are taken into account individually in the estimation of esds in distances, angles and torsion angles; correlations between esds in cell parameters are only used when they are defined by crystal symmetry. An approximate (isotropic) treatment of cell esds is used for estimating esds involving l.s. planes.
Refinement. Refinement of F^2^ against ALL reflections. The weighted R-factor wR and goodness of fit S are based on F^2^, conventional R-factors R are based on F, with F set to zero for negative F^2^. The threshold expression of F^2^ > 2sigma(F^2^) is used only for calculating R-factors(gt) etc. and is not relevant to the choice of reflections for refinement. R-factors based on F^2^ are statistically about twice as large as those based on F, and R- factors based on ALL data will be even larger. H-atoms attached to carbon were placed in calculated positions (C—H = 0.95 - 0.99 Å) and were included as riding contributions with isotropic displacement parameters 1.2 - 1.5 times those of the attached atoms. Those attached to nitrogen were placed in locations derived from a difference map and refined with a DFIX 0.91 0.01 instruction. Refined as a 2-component twin.

### Fractional atomic coordinates and isotropic or equivalent isotropic displacement parameters (Å^2^)

**Table:**

	*x*	*y*	*z*	*U*_iso_*/*U*_eq_	
O1	0.8749 (4)	0.5209 (2)	0.31078 (12)	0.0307 (6)	
O2	0.7779 (4)	0.3547 (3)	0.48140 (13)	0.0386 (7)	
O3	0.8042 (4)	0.3592 (3)	0.25076 (12)	0.0348 (7)	
O4	0.6845 (5)	−0.1158 (3)	0.23160 (17)	0.0516 (9)	
N1	0.7413 (5)	0.1528 (3)	0.42168 (15)	0.0309 (7)	
H1	0.750 (7)	0.206 (3)	0.4557 (16)	0.052 (14)*	
N2	0.4861 (5)	0.0341 (3)	0.32311 (16)	0.0325 (8)	
H2A	0.381 (5)	−0.006 (4)	0.305 (2)	0.066 (17)*	
C1	0.5648 (5)	−0.0283 (3)	0.37991 (18)	0.0293 (8)	
C2	0.5022 (6)	−0.1448 (4)	0.3935 (2)	0.0346 (9)	
H2	0.417357	−0.185984	0.361639	0.042*	
C3	0.5608 (7)	−0.2015 (4)	0.4524 (2)	0.0410 (11)	
H3	0.515597	−0.280802	0.460548	0.049*	
C4	0.6847 (7)	−0.1440 (4)	0.4995 (2)	0.0422 (11)	
H4	0.726016	−0.183596	0.539729	0.051*	
C5	0.7479 (6)	−0.0280 (4)	0.48747 (19)	0.0350 (9)	
H5	0.831745	0.012667	0.519869	0.042*	
C6	0.6895 (6)	0.0296 (3)	0.42827 (18)	0.0278 (8)	
C7	0.7802 (5)	0.2070 (3)	0.36688 (18)	0.0270 (8)	
C8	0.8001 (6)	0.1246 (4)	0.30983 (19)	0.0342 (9)	
H8A	0.873700	0.051597	0.325464	0.041*	
H8B	0.873351	0.167001	0.277963	0.041*	
C9	0.6076 (6)	0.0860 (4)	0.27573 (19)	0.0322 (9)	
H9	0.541795	0.160745	0.256997	0.039*	
C10	0.8108 (5)	0.3343 (3)	0.36710 (17)	0.0261 (8)	
C11	0.8122 (5)	0.4025 (3)	0.42765 (18)	0.0288 (8)	
C12	0.8580 (5)	0.5294 (3)	0.42500 (18)	0.0292 (8)	
H12	0.864632	0.576254	0.464103	0.035*	
C13	0.8913 (6)	0.5830 (3)	0.36913 (19)	0.0290 (8)	
C14	0.8277 (5)	0.3984 (3)	0.30673 (18)	0.0264 (8)	
C15	0.9490 (7)	0.7108 (4)	0.3614 (2)	0.0390 (10)	
H15A	0.967050	0.750057	0.404507	0.058*	
H15B	0.848667	0.753521	0.333246	0.058*	
H15C	1.069420	0.713570	0.341196	0.058*	
C16	0.6325 (6)	0.0022 (3)	0.21928 (19)	0.0305 (8)	
C17	0.6236 (6)	0.0268 (4)	0.15511 (18)	0.0382 (10)	
H17	0.594168	0.102485	0.134101	0.046*	
C18	0.6687 (7)	−0.0869 (5)	0.1245 (2)	0.0530 (14)	
H18	0.671856	−0.100553	0.078868	0.064*	
C19	0.7044 (7)	−0.1673 (5)	0.1713 (3)	0.0563 (14)	
H19	0.739039	−0.249397	0.164714	0.068*	

### Atomic displacement parameters (Å^2^)

**Table:**

	*U*^11^	*U*^22^	*U*^33^	*U*^12^	*U*^13^	*U*^23^
O1	0.0372 (16)	0.0320 (14)	0.0233 (13)	−0.0023 (12)	0.0052 (11)	0.0010 (11)
O2	0.0556 (18)	0.0400 (16)	0.0201 (13)	−0.0164 (14)	0.0036 (12)	−0.0005 (12)
O3	0.0434 (17)	0.0393 (16)	0.0213 (13)	0.0028 (13)	0.0017 (12)	−0.0026 (11)
O4	0.063 (2)	0.0425 (18)	0.052 (2)	0.0052 (16)	0.0182 (17)	0.0024 (16)
N1	0.0393 (19)	0.0303 (17)	0.0230 (16)	−0.0078 (15)	0.0023 (14)	−0.0018 (13)
N2	0.0335 (19)	0.0390 (19)	0.0253 (16)	−0.0079 (15)	0.0038 (14)	0.0006 (14)
C1	0.032 (2)	0.032 (2)	0.0263 (19)	−0.0001 (17)	0.0106 (16)	−0.0033 (16)
C2	0.040 (2)	0.031 (2)	0.034 (2)	−0.0042 (18)	0.0112 (18)	−0.0050 (17)
C3	0.057 (3)	0.028 (2)	0.042 (2)	−0.005 (2)	0.022 (2)	0.0029 (18)
C4	0.055 (3)	0.043 (2)	0.031 (2)	0.007 (2)	0.013 (2)	0.0119 (19)
C5	0.039 (2)	0.042 (2)	0.0250 (19)	0.0008 (19)	0.0062 (17)	0.0020 (17)
C6	0.034 (2)	0.0257 (18)	0.0252 (18)	−0.0027 (16)	0.0093 (15)	−0.0001 (15)
C7	0.0247 (19)	0.0323 (19)	0.0238 (18)	−0.0049 (16)	0.0020 (15)	−0.0009 (15)
C8	0.041 (2)	0.034 (2)	0.030 (2)	−0.0044 (18)	0.0093 (17)	−0.0025 (17)
C9	0.038 (2)	0.034 (2)	0.0251 (19)	−0.0018 (18)	0.0073 (16)	−0.0003 (16)
C10	0.029 (2)	0.0281 (18)	0.0213 (17)	−0.0044 (15)	0.0037 (15)	0.0008 (14)
C11	0.030 (2)	0.034 (2)	0.0218 (18)	−0.0079 (16)	0.0009 (15)	−0.0001 (15)
C12	0.036 (2)	0.0288 (19)	0.0227 (18)	−0.0055 (16)	0.0021 (15)	−0.0031 (15)
C13	0.030 (2)	0.0299 (19)	0.0277 (19)	−0.0011 (16)	0.0040 (16)	−0.0011 (15)
C14	0.0254 (19)	0.0285 (19)	0.0253 (18)	−0.0006 (15)	0.0027 (15)	0.0005 (15)
C15	0.049 (3)	0.034 (2)	0.035 (2)	−0.004 (2)	0.014 (2)	0.0023 (18)
C16	0.034 (2)	0.0289 (19)	0.0282 (19)	−0.0054 (17)	0.0044 (16)	−0.0004 (16)
C17	0.036 (2)	0.056 (3)	0.0219 (19)	0.008 (2)	0.0042 (17)	0.0102 (18)
C18	0.033 (3)	0.090 (4)	0.035 (2)	−0.003 (3)	0.003 (2)	−0.026 (3)
C19	0.048 (3)	0.047 (3)	0.078 (4)	−0.003 (2)	0.025 (3)	−0.023 (3)

### Geometric parameters (Å, º)

**Table:**

O1—C13	1.372 (4)	C7—C10	1.419 (5)
O1—C14	1.389 (4)	C7—C8	1.498 (5)
O2—C11	1.267 (4)	C8—C9	1.513 (6)
O3—C14	1.220 (4)	C8—H8A	0.9900
O4—C16	1.366 (5)	C8—H8B	0.9900
O4—C19	1.380 (6)	C9—C16	1.504 (5)
N1—C7	1.326 (5)	C9—H9	1.0000
N1—C6	1.415 (5)	C10—C14	1.440 (5)
N1—H1	0.912 (12)	C10—C11	1.449 (5)
N2—C1	1.412 (5)	C11—C12	1.436 (5)
N2—C9	1.474 (5)	C12—C13	1.331 (5)
N2—H2A	0.906 (12)	C12—H12	0.9500
C1—C2	1.393 (5)	C13—C15	1.477 (5)
C1—C6	1.402 (5)	C15—H15A	0.9800
C2—C3	1.382 (6)	C15—H15B	0.9800
C2—H2	0.9500	C15—H15C	0.9800
C3—C4	1.381 (6)	C16—C17	1.338 (5)
C3—H3	0.9500	C17—C18	1.451 (7)
C4—C5	1.383 (6)	C17—H17	0.9500
C4—H4	0.9500	C18—C19	1.309 (7)
C5—C6	1.391 (5)	C18—H18	0.9500
C5—H5	0.9500	C19—H19	0.9500
			
O2···C3^i^	3.322 (5)	C3···C15^v^	3.591 (6)
O2···N1	2.537 (4)	C4···C6^i^	3.387 (5)
O2···C12^ii^	3.282 (4)	C10···C18^iii^	3.497 (6)
O3···C9	3.372 (4)	C11···C17^iii^	3.600 (5)
O3···C8	2.856 (4)	C11···C18^iii^	3.428 (5)
O3···N2^iii^	3.079 (4)	C12···C12^ii^	3.541 (5)
O4···N2	2.955 (5)	C12···C17^iii^	3.589 (5)
O4···C1	3.376 (5)	C14···C16^iii^	3.407 (5)
O1···H2A^iii^	2.84 (4)	C1···H8A	2.68
O2···H12^ii^	2.74	C2···H17^vi^	2.91
O2···H3^i^	2.62	C3···H17^vi^	2.93
O2···H1	1.72 (3)	C6···H8A	2.59
O3···H9	2.87	C11···H1	2.28 (3)
O3···H2A^iii^	2.20 (4)	C14···H2A^iii^	2.79 (4)
H15C···O3^iv^	2.70	C14···H8B	2.64
O3···H2^iii^	2.68	H2···H2A	2.29
O3···H8B	2.23	H2···H17^vi^	2.33
O4···H8A	2.88	H3···H17^vi^	2.38
O4···H15B^v^	2.70	H5···H1	2.55
N1···N2	2.865 (4)	H12···H15A	2.42
N2···H19^iii^	2.89		
			
C13—O1—C14	122.3 (3)	C16—C9—C8	110.8 (3)
C16—O4—C19	106.0 (4)	N2—C9—H9	107.3
C7—N1—C6	126.2 (3)	C16—C9—H9	107.3
C7—N1—H1	111 (3)	C8—C9—H9	107.3
C6—N1—H1	123 (3)	C7—C10—C14	120.5 (3)
C1—N2—C9	122.0 (3)	C7—C10—C11	120.1 (3)
C1—N2—H2A	109 (3)	C14—C10—C11	119.2 (3)
C9—N2—H2A	115 (3)	O2—C11—C12	120.1 (3)
C2—C1—C6	117.6 (4)	O2—C11—C10	123.0 (3)
C2—C1—N2	120.6 (4)	C12—C11—C10	116.9 (3)
C6—C1—N2	121.3 (3)	C13—C12—C11	121.7 (3)
C3—C2—C1	121.4 (4)	C13—C12—H12	119.2
C3—C2—H2	119.3	C11—C12—H12	119.2
C1—C2—H2	119.3	C12—C13—O1	121.5 (3)
C4—C3—C2	120.6 (4)	C12—C13—C15	126.2 (4)
C4—C3—H3	119.7	O1—C13—C15	112.3 (3)
C2—C3—H3	119.7	O3—C14—O1	114.0 (3)
C3—C4—C5	119.3 (4)	O3—C14—C10	128.3 (3)
C3—C4—H4	120.4	O1—C14—C10	117.7 (3)
C5—C4—H4	120.4	C13—C15—H15A	109.5
C4—C5—C6	120.5 (4)	C13—C15—H15B	109.5
C4—C5—H5	119.8	H15A—C15—H15B	109.5
C6—C5—H5	119.8	C13—C15—H15C	109.5
C5—C6—C1	120.8 (4)	H15A—C15—H15C	109.5
C5—C6—N1	117.8 (3)	H15B—C15—H15C	109.5
C1—C6—N1	121.1 (3)	C17—C16—O4	111.0 (4)
N1—C7—C10	119.1 (3)	C17—C16—C9	129.4 (4)
N1—C7—C8	115.7 (3)	O4—C16—C9	119.5 (3)
C10—C7—C8	125.0 (3)	C16—C17—C18	105.1 (4)
C7—C8—C9	112.2 (3)	C16—C17—H17	127.4
C7—C8—H8A	109.2	C18—C17—H17	127.4
C9—C8—H8A	109.2	C19—C18—C17	107.5 (4)
C7—C8—H8B	109.2	C19—C18—H18	126.3
C9—C8—H8B	109.2	C17—C18—H18	126.3
H8A—C8—H8B	107.9	C18—C19—O4	110.4 (4)
N2—C9—C16	113.1 (3)	C18—C19—H19	124.8
N2—C9—C8	110.8 (3)	O4—C19—H19	124.8
			
C9—N2—C1—C2	−126.6 (4)	C7—C10—C11—O2	−3.5 (6)
C9—N2—C1—C6	61.5 (5)	C14—C10—C11—O2	172.3 (4)
C6—C1—C2—C3	−0.2 (6)	C7—C10—C11—C12	175.6 (3)
N2—C1—C2—C3	−172.4 (4)	C14—C10—C11—C12	−8.6 (5)
C1—C2—C3—C4	−0.3 (6)	O2—C11—C12—C13	−178.7 (4)
C2—C3—C4—C5	0.8 (7)	C10—C11—C12—C13	2.2 (6)
C3—C4—C5—C6	−0.8 (6)	C11—C12—C13—O1	3.1 (6)
C4—C5—C6—C1	0.4 (6)	C11—C12—C13—C15	−176.7 (4)
C4—C5—C6—N1	173.1 (4)	C14—O1—C13—C12	−1.7 (6)
C2—C1—C6—C5	0.1 (5)	C14—O1—C13—C15	178.1 (3)
N2—C1—C6—C5	172.2 (4)	C13—O1—C14—O3	174.5 (3)
C2—C1—C6—N1	−172.3 (3)	C13—O1—C14—C10	−4.9 (5)
N2—C1—C6—N1	−0.2 (5)	C7—C10—C14—O3	6.4 (6)
C7—N1—C6—C5	147.3 (4)	C11—C10—C14—O3	−169.4 (4)
C7—N1—C6—C1	−40.0 (6)	C7—C10—C14—O1	−174.3 (3)
C6—N1—C7—C10	173.6 (4)	C11—C10—C14—O1	9.9 (5)
C6—N1—C7—C8	−10.1 (6)	C19—O4—C16—C17	−1.4 (5)
N1—C7—C8—C9	79.4 (4)	C19—O4—C16—C9	−177.3 (4)
C10—C7—C8—C9	−104.5 (4)	N2—C9—C16—C17	135.7 (4)
C1—N2—C9—C16	94.4 (4)	C8—C9—C16—C17	−99.2 (5)
C1—N2—C9—C8	−30.7 (5)	N2—C9—C16—O4	−49.3 (5)
C7—C8—C9—N2	−52.9 (4)	C8—C9—C16—O4	75.8 (5)
C7—C8—C9—C16	−179.3 (3)	O4—C16—C17—C18	1.8 (5)
N1—C7—C10—C14	−171.6 (4)	C9—C16—C17—C18	177.1 (4)
C8—C7—C10—C14	12.4 (6)	C16—C17—C18—C19	−1.4 (5)
N1—C7—C10—C11	4.1 (6)	C17—C18—C19—O4	0.6 (6)
C8—C7—C10—C11	−171.9 (4)	C16—O4—C19—C18	0.5 (5)

Symmetry codes: (i) −*x*+1, −*y*, −*z*+1; (ii) −*x*+2, −*y*+1, −*z*+1; (iii) −*x*+1, *y*+1/2, −*z*+1/2; (iv) −*x*+2, *y*+1/2, −*z*+1/2; (v) *x*, *y*−1, *z*; (vi) −*x*+1, *y*−1/2, −*z*+1/2.

### Hydrogen-bond geometry (Å, º)

**Table:**

*D*—H···*A*	*D*—H	H···*A*	*D*···*A*	*D*—H···*A*
N1—H1···O2	0.91 (1)	1.72 (3)	2.538 (4)	148 (4)
N2—H2*A*···O3^vi^	0.91 (1)	2.20 (2)	3.079 (4)	162 (5)

Symmetry code: (vi) −*x*+1, *y*−1/2, −*z*+1/2.
